# Dimethyl fumarate ameliorated pyroptosis in contrast-induced acute renal injury by regulating endoplasmic reticulum stress and JAK2-STAT3 pathway

**DOI:** 10.1080/0886022X.2025.2504633

**Published:** 2025-05-19

**Authors:** Xiaoning An, Min Yin, Yilan Shen, Xieyi Guo, Youhua Xu, Dongsheng Cheng, Dingkun Gui

**Affiliations:** a Department of Nephrology, Shanghai Sixth People’s Hospital Affiliated to Shanghai Jiao Tong University School of Medicine, Shanghai, China; b Department of Traditional Chinese Medicine, Shanghai Pudong New Area People’s Hospital, Shanghai, China; c State Key Laboratory of Quality Research in Chinese Medicine, Macau University of Science and Technology, Macao, China

**Keywords:** Contrast-induced acute renal injury, dimethyl fumarate, endoplasmic reticulum stress, JAK2–STAT3 pathway, pyroptosis

## Abstract

**Background:**

Inflammation and oxidative stress are important pathological processes of contrast-induced acute renal injury (CIAKI). This study explored whether DMF had therapeutic effects and investigated the underlying mechanism in CIAKI.

**Methods:**

A CIAKI animal model was established in C57BL/6J mice with iohexol, and DMF was used as an intervention. *In vitro*, HK-2 cells were treated with iohexol and DMF. RNA-seq analysis was performed on the renal tissue of the mice. Protein–protein interaction (PPI), and enrichment analysis were subsequently conducted. In addition, endoplasmic reticulum stress (ERS) activation and STAT3 inhibition were used to study the relationships among ERS, the JAK2–STAT3 pathway and pyroptosis.

**Results:**

DMF improved the renal function of CIAKI model mice. Enrichment analysis revealed that the differentially expressed genes (DEGs) were enriched mostly in the acute phase response and the JAK–STAT pathway. The results revealed that inflammation, ERS and pyroptosis increased in the CIAKI group but decreased after DMF treatment. Further study revealed that the JAK2–STAT3 pathway was overactivated *in vivo* and *in vitro* and that DMF inhibited the JAK2–STAT3 pathway. In addition, ERS activation could increase the JAK2–STAT3 pathway and pyroptosis, while STAT3 knockdown could reverse pyroptosis, indicating that ERS could activate the JAK2–STAT3 pathway, further triggering pyroptosis. DMF ameliorated pyroptosis through regulating ERS and the JAK2–STAT3 pathway in CIAKI.

**Conclusion:**

This study demonstrated that DMF had renoprotective effects on CIAKI. DMF ameliorated pyroptosis through the inhibition of ERS and the JAK2–STAT3 pathway.

## 1. Introduction

Acute kidney injury (AKI) is a serious global health concern, accounting for approximately 2 million deaths annually, and is associated with high morbidity and mortality rates [1,2]. The widespread use of iodinated radiocontrast media (RCM) in clinical diagnostics has made contrast-induced acute kidney injury (CIAKI) the third leading cause of hospital-acquired AKI, significantly contributing to in-hospital mortality and prolonged hospitalization [3]. Current preventive strategies for CIAKI primarily target renal vasoconstriction, tubular acidification, and hypoxia-induced oxidative stress. Although preprocedural intravenous hydration is routinely administered, its efficacy remains inconclusive, and the outcomes of vasodilating agents are inconsistent [4,5]. These limitations highlight the need for novel therapeutic strategies.

Dimethyl fumarate (DMF), an FDA-approved oral treatment for multiple sclerosis (MS), has demonstrated broad therapeutic potential because of its antioxidant, immunomodulatory, neuroprotective, anti-inflammatory, and antiproliferative properties [6]. Studies have shown that DMF can play a protective role in many kidney diseases. DMF can attenuate lipopolysaccharide-induced septic AKI by suppressing NF-κB p65 phosphorylation and macrophage activation [7]. DMF has dual reno- and neuro-protective effects on uremic encephalopathy in a renal ischemia/reperfusion model [8]. DMF has been shown to exert therapeutic effects on various types of AKI by mitigating inflammation and oxidative stress. However, whether DMF has therapeutic potential in CIAKI remains unexplored. Therefore, we investigated the renoprotective effect of DMF on CIAKI.

Increasing evidence indicates that pyroptosis is an important pathological process in CIAKI [9–11]. Some drugs, such as klotho and baicalin, can improve the renal function of CIAKI patients by inhibiting pyroptosis [9,12]. DMF can play a therapeutic role in several diseases by inhibiting pyroptosis, such as nerve injury and autoimmune hepatitis [13,14]. However, whether DMF can ameliorate pyroptosis in CIAKI is unknown. Studies have demonstrated that the Janus kinase/signal transduction and transcription activator (JAK–STAT) pathway is involved in various kidney diseases [15]. In focal segmental glomerulosclerosis (FSGS), the JAK–STAT signaling pathway is activated, with increased phosphorylation of STAT1 and STAT3 in both the glomerulus and renal tubulointerstitium [16]. In a unilateral ureteral obstruction model, STAT3 was activated, while a STAT3 inhibitor inhibited the activation and fibrosis of renal interstitial fibroblasts, indicating that the increase in STAT3 activity mediated the activation of renal interstitial fibroblasts and the progression of renal fibrosis [17]. However, the role of the JAK–STAT pathway in CIAKI has not yet been reported. Studies have shown that RCM can induce the expression of endoplasmic reticulum stress (ERS) markers such as GRP78 and CHOP in the kidney and that the inhibition of ERS by drugs can alleviate AKI induced by RCM [18–20]. Studies have shown that DMF can improve ERS. DMF can ameliorate ERS in the striatum of Huntington rats through the IRE1α/JNK and PERK/CHOP pathways [21]. However, whether DMF can improve ERS in CIAKI is still unknown.

Therefore, in this study, we explored whether DMF has a therapeutic effect on CIAKI and investigated the underlying mechanism.

## 2. Methods

### 2.1. Animals and CIAKI model

C57BL/6 mice were purchased from Shanghai Sippe-Bk Lab Animal Co., Ltd. The mice were housed in the animal facility of Shanghai Sixth People’s Hospital according to relevant regulations, and all animal experiments were approved by the Animal Care Committee of our institution.

Eight- to ten-week-old adult male WT mice were randomly divided into the control group (Control; *n* = 6), iohexol group (CIAKI; *n* = 6), iohexol + DMF low-dose group (C.D.L.; *n* = 5), iohexol + DMF medium-dose group (C.D.M.; *n* = 5) and iohexol + DMF high-dose group (C.D.H.; *n* = 6). The dosage of iohexol in the mice was 4 mg/g, and the dosages of DMF in the C.D.L., C.D.M. and C.D.H. groups were 10 mg/g, 20 mg/g and 40 mg/g, respectively. First, the mice were pretreated with DMF orally for 7 days. After 16 h of water deprivation, indomethacin (Sigma, 10 mg/kg) and L-NAME (Sigma, 10 mg/kg) were injected intraperitoneally into the mice to inhibit the synthesis of prostaglandin and nitric oxide, followed by the intraperitoneal injection of iohexol (Sigma). The control mice were injected with saline. The interval of intraperitoneal injection was 15 min. All the mice were sacrificed after 24 h of iohexol/saline administration. The serum and kidney were harvested for further analysis.

### 2.2. Cell culture and treatment

Human immortalized proximal tubular (HK-2) cells were purchased from the Cell Bank/Stem Cell Bank, Chinese Academy of Sciences. The cells were grown in DMEM/F12 with a final concentration of 10% fetal bovine serum (FBS) and 1% penicillin/streptomycin at 37 °C with 5% CO_2_.

Iohexol was selected to establish a CIAKI cell model. In most studies, the iodine content is commonly used as the unit of measurement. In this study, the final iodine concentration in the cell culture medium was set at 50 mgI/mL. HK-2 cells were plated in six-well plates and pretreated with DMF at low (10 μM), medium (20 μM) or high (40 μM) concentrations. One hour later, the cells were treated with iohexol for 48 h. The cells were harvested for further analysis.

To investigate the effects of STAT3 signaling in CIAKI, siRNA was used to knockdown the STAT3 gene. siRNAs and liposome RNAiMAX were mixed, and the transfection mixture was subsequently incubated at 37 °C for 30 min. Subsequently, siRNA gene transfection was performed by incubating HK-2 cells with medium containing the transfection mixture for 6 h. The transfection mixture was subsequently replaced with fresh cell culture medium.

HK-2 cells were treated with siRNA-STAT3 or siRNA-STAT3 plus DMF (40 μM). One hour later, HK-2 cells were treated with iohexol for 48 h. The cells were harvested for further analysis. To study the role of ERS in CIAKI, HK-2 cells were treated with the ERS inducer tunicamycin (20 nM). First, HK-2 cells were pretreated with DMF (40 μM). One hour later, the cells were treated with iohexol or iohexol plus tunicamycin (20 nM) for 48 h. To study the relationship between ERS and STAT3 signaling, HK-2 cells were pretreated with siRNA-STAT3 or siRNA-STAT3 plus DMF (40 μM). One hour later, the cells were treated with iohexol or iohexol plus tunicamycin (20 nM) for 48 h. The cells were harvested for further analysis.

### 2.3. Biochemical analysis

Blood urea nitrogen (BUN), serum creatinine (Scr), malondialdehyde (MDA), and superoxide dismutase (SOD) levels were assessed with assay kits (Nanjing Jiancheng, Nanjing, China). The experimental steps were performed according to the manufacturer’s instructions.

### 2.4. HE and immunohistochemistry staining

For HE staining, the slides were deparaffinized, hydrated, and then stained with hematoxylin for 3 min and eosin for 60 s. Finally, the slides were covered with neutral resin and observed under a microscope.

For immunohistochemistry (IHC) staining, the slides were deparaffinized, hydrated, and then treated with 3% H_2_O_2_ for 10 min. After heat retrieval and blocking with 1% BSA, the slides were incubated with anti-NGAL antibody (CST) and anti-F4/80 antibody (CST) followed by horseradish peroxidase (HRP)-labeled secondary antibodies (Jackson ImmunoResearch). The slides were then covered with diaminobenzidine (DAB), counterstained with hematoxylin and observed under a microscope.

### 2.5. RNA-seq analysis

The services of Suzhou Bionovogene were used for RNA-seq analysis. Differentially expressed genes (DEGs) were identified on the basis of the following criteria: |log2-fold change (FC)| > 1.2 and a false discovery rate (FDR) < vv0.05. The outcomes of the DEGs were uploaded to the STRING database for protein–protein interaction (PPI) analysis, resulting in the construction of an interaction network. The interaction network was imported into the Cytoscape software to filter the degrees of freedom of nodes in the network to obtain hub genes. The hub genes were subsequently analyzed using the Gene Ontology (GO) and Kyoto Encyclopedia of Genes and Genomes (KEGG) pathway enrichment analyses.

### 2.6. Enzyme-linked immunosorbent assay (ELISA)

The concentrations of kidney injury molecule-1 (KIM-1), neutrophil gelatinase-associated lipocalin (NGAL), interleukin-6 (IL-6), tumor necrosis factor-α (TNF-α), interleukin-1β (IL-1β), and interleukin-18 (IL-18) in the serum of the mice were determined using ELISA kits according to the manufacturer’s instructions. The absorbance of the samples at a wavelength of 450 nm was measured with a BioTek microplate reader.

### 2.7. ROS detection

Dihydroethidium was used as a fluorescent probe to measure the production of ROS. Mouse frozen sections and HK-2 cells were incubated with FBS-free medium containing 5 μM dihydroethidium at 37 °C for approximately 30 min to load fluorescent probes, counterstained with Hoechst 33342 and observed under a fluorescence microscope.

### 2.8. Western blot

RIPA lysis buffer was used to extract protein from kidney tissue and HK-2 cells, and the cell lysates were separated by SDS–PAGE and then transferred to PVDF membranes. After blocking with 5% BSA, the PVDF membranes were incubated with anti-β-actin antibody (CST), anti-PERK antibody (CST), anti-p-eIF2α antibody (CST), anti-ATF4 antibody (CST), anti-CHOP antibody (CST), anti-NLRP3 antibody (ABclonal), anti-caspase 1 antibody (CST), anti-GSDMD antibody (CST), anti-cleaved GSDMD antibody (ABclonal), anti-ASC antibody (ABclonal), anti-JAK antibody (CST), anti-phospho JAK2 antibody (ABclonal), anti-STAT3 antibody (CST) and anti-phospho STAT3 antibody (ABclonal) overnight at 4 °C. The membranes were subsequently incubated with the appropriate HRP-labeled secondary antibodies. Proteins were finally visualized by ECL, and ImageJ software was used to observe the band intensity. Standardization was performed using β-actin.

### 2.9. Quantitative real-time PCR

Total RNA was extracted from mouse kidney tissue and HK-2 cells with TRIzol reagent (Invitrogen). The RNA from each sample was converted to cDNA using a reverse transcription kit (Takara). Quantitative real-time PCR (qRT–PCR) was performed using a standard SYBR Green PCR kit (Takara) to measure the levels of the GAPDH, IL–6 and TNF–α mRNAs. The relative amount of mRNA was normalized to that of GAPDH and calculated using the delta–delta method from threshold cycle numbers. The sequences of primers used were designed and synthesized by Biotech, Shanghai (Table S1).

## 3. Statistical analysis

The data are expressed as the means ± SEMs. One-way ANOVA and the Kruskal–Wallis test were used to determine the statistical significance of differences between groups. Data were considered statistically significant if *p* < 0.05. All the statistical analyses were performed using GraphPad software version 7.0.

## 4. Results

### 4.1. DMF ameliorated contrast-induced renal injury in mice

**Figure 1.**
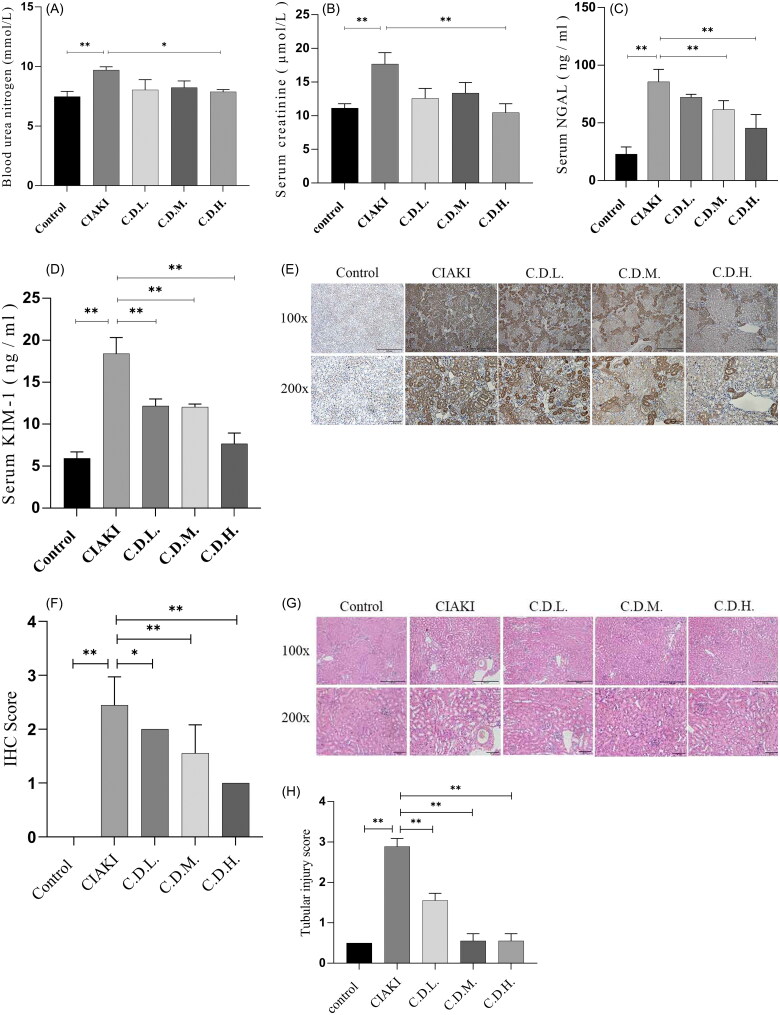
DMF ameliorated contrast-induced renal injury in mice. (A) Summarized serum BUN levels, (B) Summarized serum Scr levels. (C) Summarized serum NGAL levels. (D) Summarized serum KIM-1 levels. (E,F) Representative immunohistochemical images and statistical assessment of NGAL in the kidney sections of mice. (G) Representative HE images in the kidney sections of mice. (H) Summarized tubular damage scores. *n* = 5–6. *p < 0.05, **p < 0.01.

To evaluate the renal function of CIAKI mice, the concentrations of BUN, Scr, NGAL and KIM-1 in the serum were detected. Compared with that of the control group, the renal function of CIAKI mice was obviously impaired. After DMF administration, the concentrations of BUN, Scr, NGAL and KIM-1 were significantly increased **(**Figure 1(A–D)**)**. IHC results revealed that the expression of NGAL increased in the CIAKI group but decreased in the DMF group **(**Figure 1(E,F)). HE staining revealed significant pathological changes, including renal tubular dilatation, cell swelling, cell necrosis, epithelial cell exfoliation and inflammatory cell infiltration, in the renal tubules of CIAKI mice. However, the DMF group presented lower renal tubular injury scores than did the CIAKI group (*p* < 0.05, Figure 1(G,H)**)**. These results provide convincing evidence for the successful establishment of a CIAKI model and underscore the potential of DMF to prevent renal dysfunction in CIAKI model mice.

### 4.2. RNA-seq analysis of renal tissues

RNA-seq analysis revealed 3163 DEGs between the CIAKI group and the control group, 1369 of which were upregulated and 1794 of which were downregulated. There were 1603 DEGs between the C.D.H. group and the CIAKI group, of which 909 DEGs were upregulated and 694 DEGs were downregulated (Figure 2(A,B)). According to the criteria |log2-fold change (FC)| > 1.2 and *p* < 0.05, the DEGs upregulated in the CIAKI group and downregulated in the C.D.H group were selected, and 87 DEGs were obtained by Venn analysis, as shown in the heatmap (Figure 2(C,D)). The proteins encoded by the DEGs were imported into the STRING database to construct a PPI network, and hub genes were obtained (Figure 2(E)). GO analysis of the top 40 hub genes revealed several genes involved in acute-phase response (*ORM1, ITIH4, IL1RN, SAA1, SAA2, SAA4*) and the inflammatory response (*CXCL2, CXCL5, CD14, IL1RN, IL2RA*). KEGG pathway analysis revealed that the hub genes were enriched mostly in the JAK–STAT pathway (Figure 2(F,G)). These results indicate that DMF might be involved in regulating acute inflammation and the JAK–STAT pathway in CIAKI.

Figure 2.RNA-seq analysis of renal tissue. Differential expression analysis of RNA-seq data. (A) Volcano plot of statistically significant DEGs at *p* < 0.05 for the CIAKI versus control group and (B) the C.D.H. group versus CIAKI group. (C) Venn diagrams showing the up-regulated DEGs identified from the CIAKI versus control groups and down-regulated DEGs identified from the C.D.H. group versus CIAKI groups at *p* < 0.05 with an absolute value of log2FoldChange ≥1.2. (D) Heat map showed 87 DEGs analyzed by Venn diagrams. (E) Top 40 hub genes of Protein-protein interaction analysis with DEGs. (F) GO analysis of top 40 hub genes. (G) KEGG analysis of top 40 hub genes.

**Figure:**
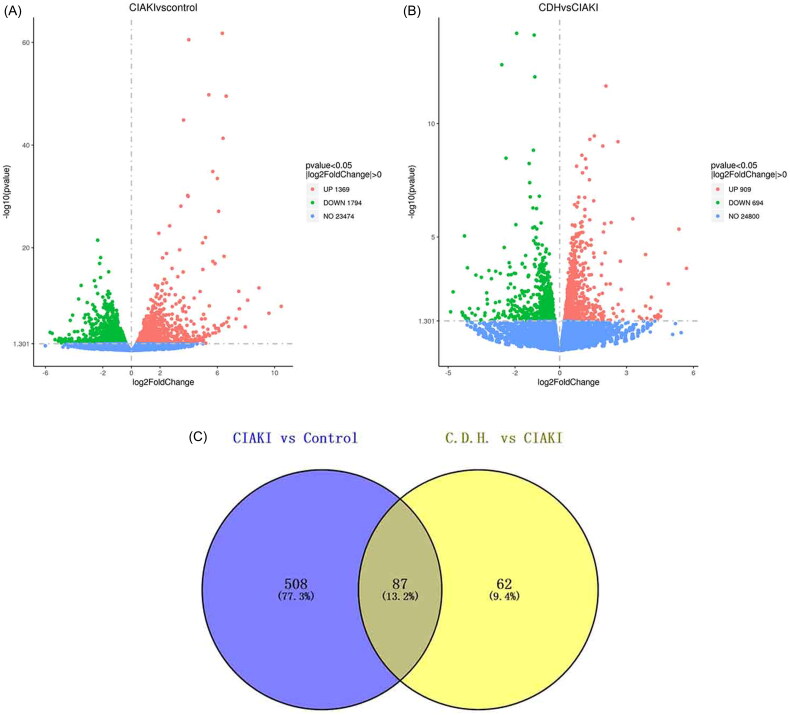

**Figure:**
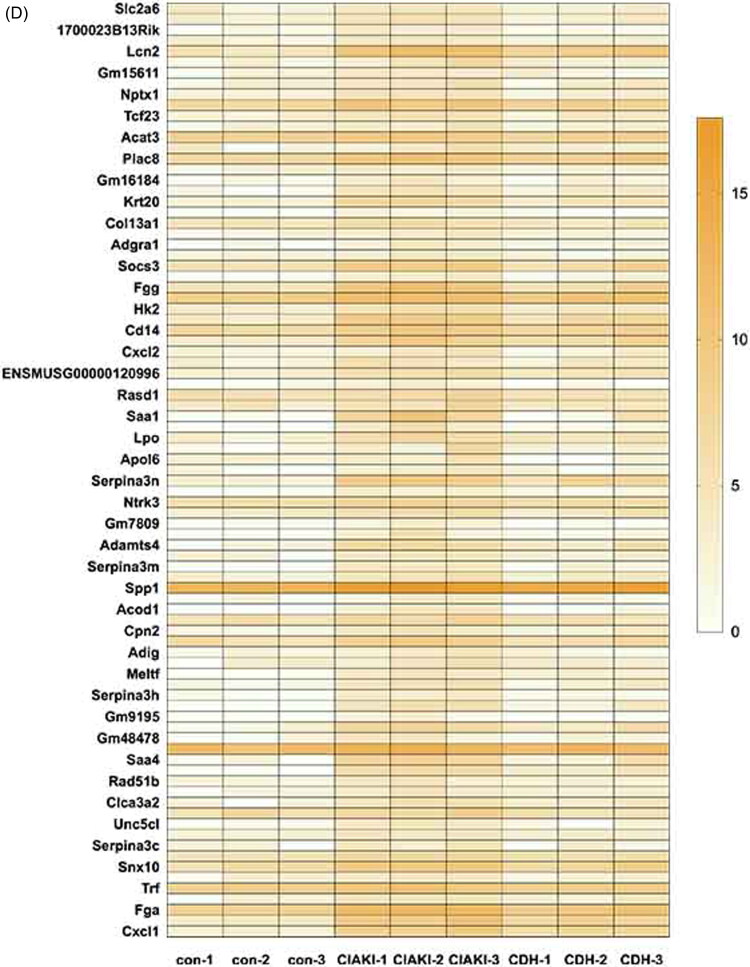

**Figure:**
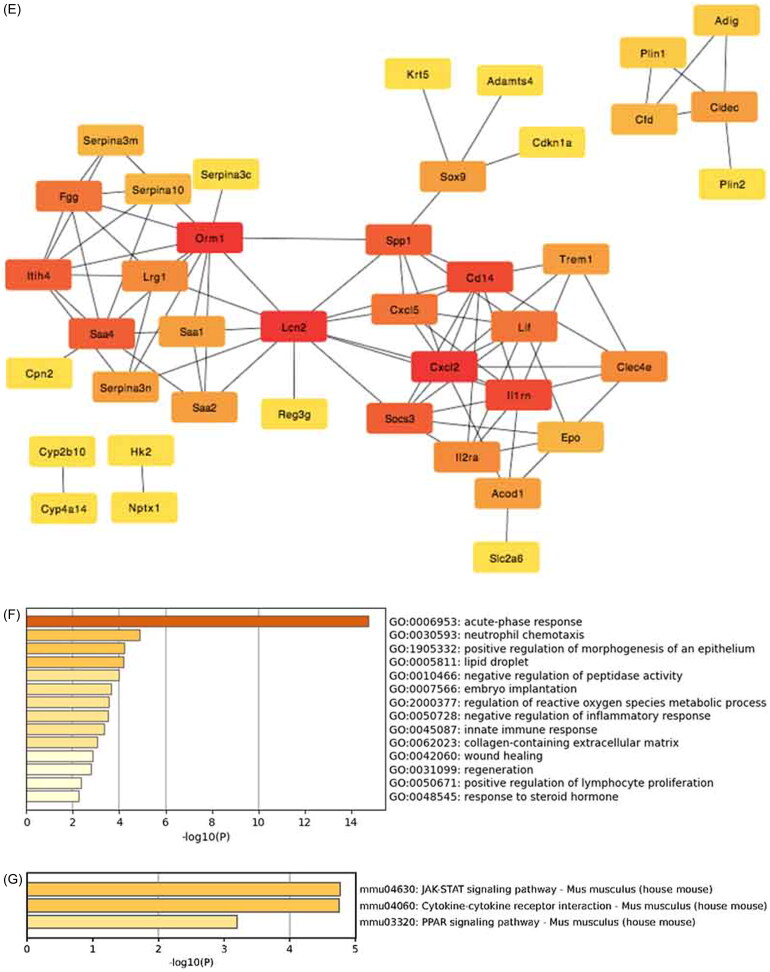

### 4.3. DMF ameliorated inflammation in contrast-induced acute kidney injury in mice and HK-2 cells

**Figure 3.**
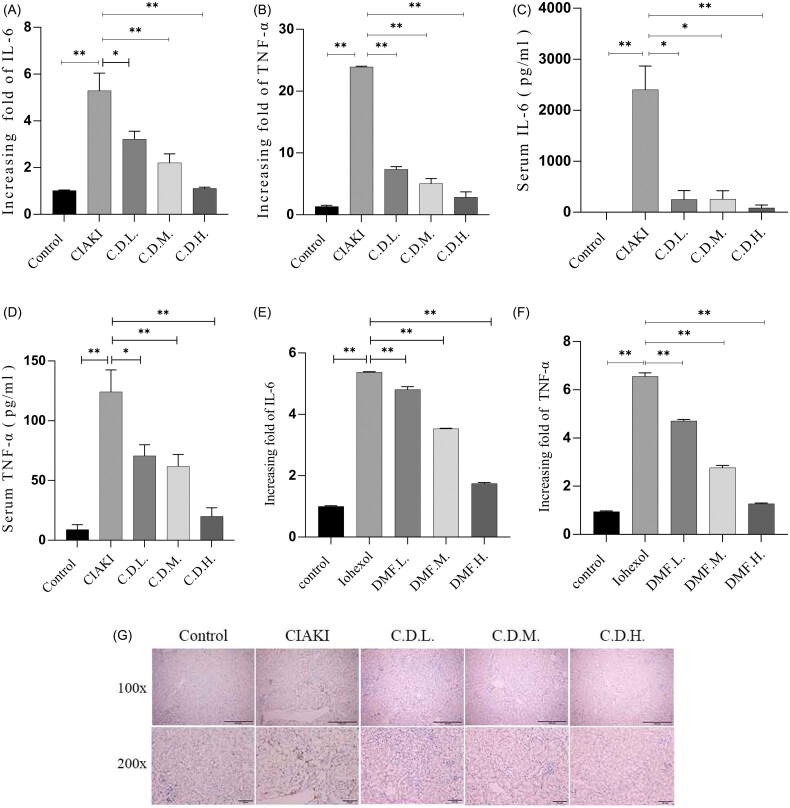
DMF ameliorated inflammation of contrast-induced acute kidney injury in mice and HK-2 cells. (A) Summarized data of IL-6 mRNA expression in the renal of mice. (B) Summarized data of TNF-α mRNA expression in the renal of mice. (C) Summarized serum IL-6 levels and (D) serum TNF-α levels in mice. (E) Summarized data of IL-6 and (F) TNF-α mRNA expression in iohexol-induced HK-2 cells with or without DMF. (G) Representative IHC image showing F4/80 protein localization. *n* = 3–6. *p < 0.05, **p < 0.01.

GO analysis revealed that there was an acute phase response in CIAKI. To evaluate whether DMF has an anti-inflammatory effect on CIAKI, proinflammatory cytokines, including IL-6 and TNF-α, were detected *via* qRT–PCR and ELISA. Compared with those in the control group, the mRNA levels of IL-6 and TNF-α in the CIAKI group were greater, whereas the level of DMF was lower (*p* < 0.05, Figure 3(A,B)). The ELISA results revealed that the concentrations of serum IL-6 and TNF-α in the CIAKI group increased but decreased in the DMF group (*p* < 0.05, Figure 3(C,D)). In addition, the IHC results revealed that DMF significantly reduced the infiltration of macrophages in the kidney tissue of the CIAKI group (Figure 3(G)). In HK-2 cells, the mRNA levels of IL-6 and TNF-α were increased by iohexol but decreased in the DMF groups (*p* < 0.05, Figure 3(E,F)). These results suggest that DMF can alleviate the inflammatory response in CIAKI.

### 4.4. DMF ameliorated pyroptosis in contrast-induced acute kidney injury in mice and HK-2 cells

Excessive pyroptosis causes cytokine storms and detrimental inflammation. To investigate whether pyroptosis was activated and whether DMF had a therapeutic effect on pyroptosis in CIAKI, the concentrations of serum IL-1β and IL-18 and the protein expression levels of NLRP3, caspase-1, GSDMD, cleaved GSDMD and ASC were evaluated by ELISA and western blotting. Compared with those in the control group, the concentrations of IL-1β and IL-18 in the serum of the CIAKI group increased significantly and decreased after DMF treatment (*p* < 0.05, Figure 4(A,B)). Western blot analysis revealed that the protein markers of pyroptosis were increased in the CIAKI group, and these increases were partially reversed by DMF treatment (*p* < 0.05, Figure 4(C–H)). The ELISA results revealed that the concentrations of IL-1β and IL-18 in the culture supernatant of HK-2 cells exposed to iohexol increased significantly and were reduced after DMF treatment (*p* < 0.05, Figure 4(I,J)). Moreover, western blot analysis revealed that the levels of pyroptosis-associated protein markers increased and that DMF reduced the levels (*p* < 0.05, Figure 4(K–P)).

Figure 4.DMF ameliorated pyroptosis of contrast-induced acute kidney injury in mice and HK-2 cells. (A) Summarized serum IL-1β and (B) IL-18 concentration in mice. (C–H) Representative Western blot images and statistical assessment of NLRP3, caspase 1, GSDMD, cleaved GSDMD and ASC in mice. (I)Summarized IL-1β and (J) IL-18 concentration in culture supernatant of iohexol-induced HK-2 cells with or without DMF. (K–P) Representative Western blot images and statistical assessment of NLRP3, caspase 1, GSDMD, cleaved GSDMD and ASC in iohexol-induced HK-2 cells with or without DMF. *n* = 3–6. *p < 0.05, **p < 0.01.

**Figure:**
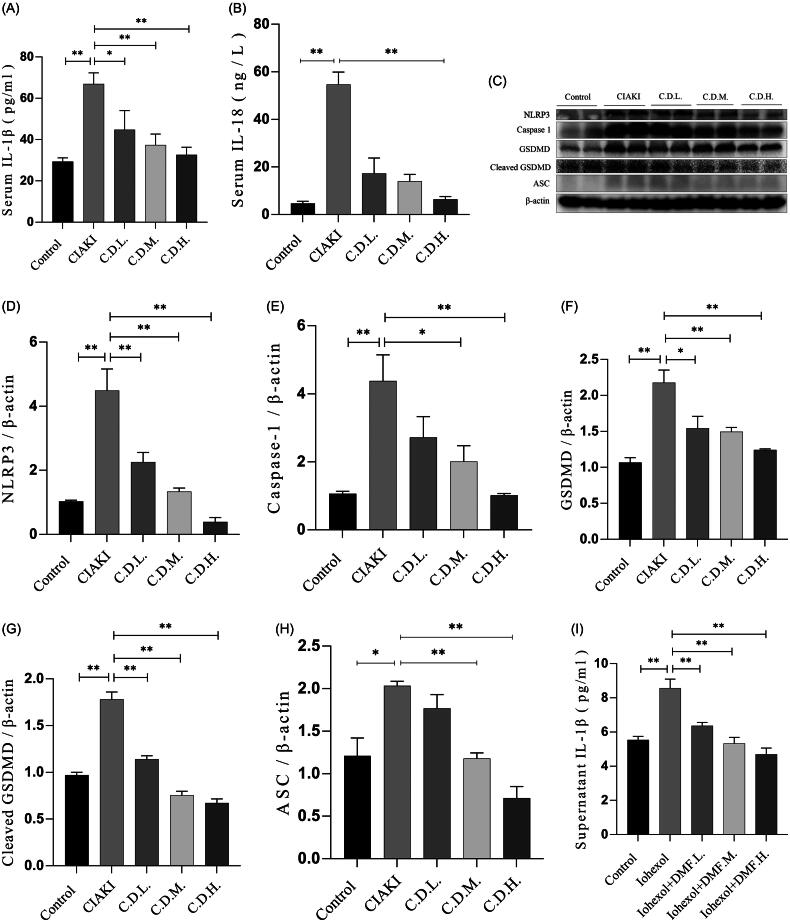

**Figure:**
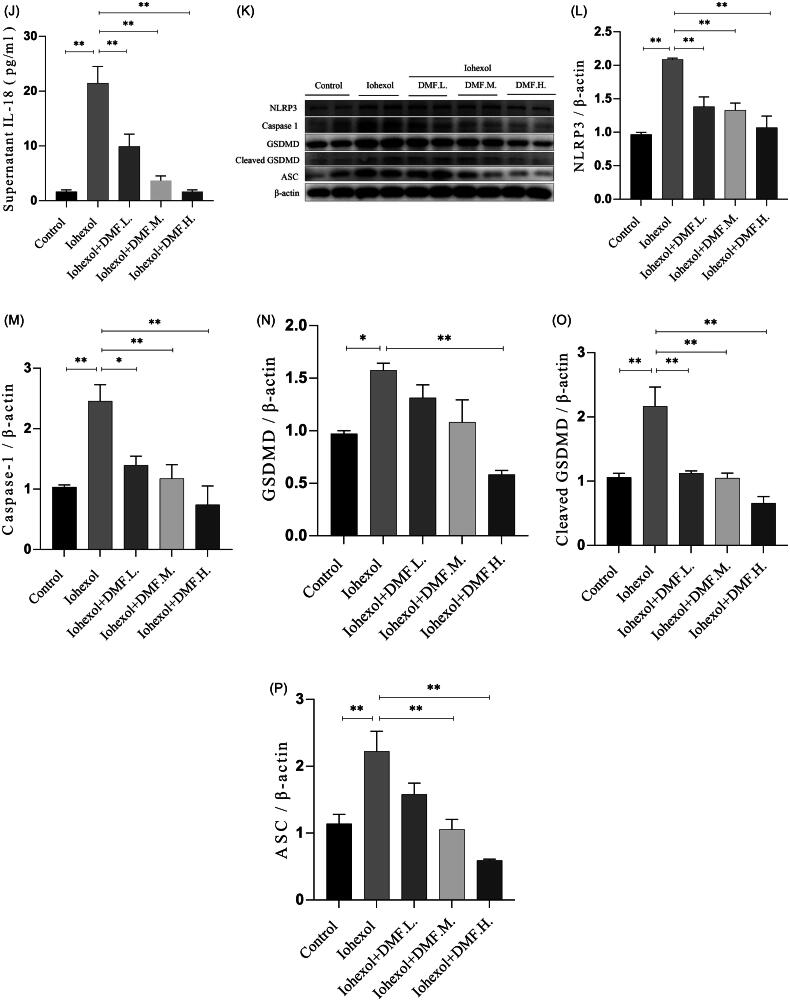

### 4.5. DMF ameliorated oxidative stress in contrast-induced acute kidney injury in mice and HK-2 cells

Oxidative stress is a key mechanism involved in the development of CIAKI. MDA and SOD were used as indicators of oxidative stress. The results of the biochemical analysis revealed that the serum MDA concentration increased and the serum SOD activity decreased in the CIAKI group, whereas the DMF treatment significantly reversed these two trends (*p* < 0.05, Figure 5(A,B)). A DHE probe was used to detect ROS production in the kidney. The results revealed that the ROS content in the CIAKI group increased and that DMF reduced the accumulation of ROS (Figure 5(C,J)). In addition, the protein expression of ERS markers was detected by western blotting. The results revealed that the protein levels of PERK, p-eIF2α, ATF4 and CHOP in the CIAKI group were increased and that DMF reduced their expression levels (*p* < 0.05, Figure 5(E–I)). In HK-2 cells, DHE staining revealed that the ROS content increased after iohexol exposure and that DMF reduced the accumulation of ROS (Figure 5(D,P)). Western blot analysis revealed that the expression of ERS protein markers was increased by iohexol, and the expression levels were decreased after DMF treatment (*p* < 0.05, Figure 5(K–O)).

Figure 5.DMF ameliorated oxidative stress of contrast-induced acute kidney injury in mice and HK-2 cells. (A) Summarized serum MDA levels and (B) serum SOD activity in mice. (C,J) Representative images and statistical assessment of ROS level in mice (200×). (E–I) representative Western blot images and statistical assessment of PERK, p-eIF2α, ATF4 and CHOP in mice. (D,P) Representative images and statistical assessment of ROS level in iohexol-induced HK-2 cells with or without DMF (200×). (K–O) representative Western blot images and statistical assessment of PERK, p-eIF2α, ATF4 and CHOP in iohexol-induced HK-2 cells with or without DMF. *n* = 3–6. *p < 0.05, **p < 0.01.

**Figure:**
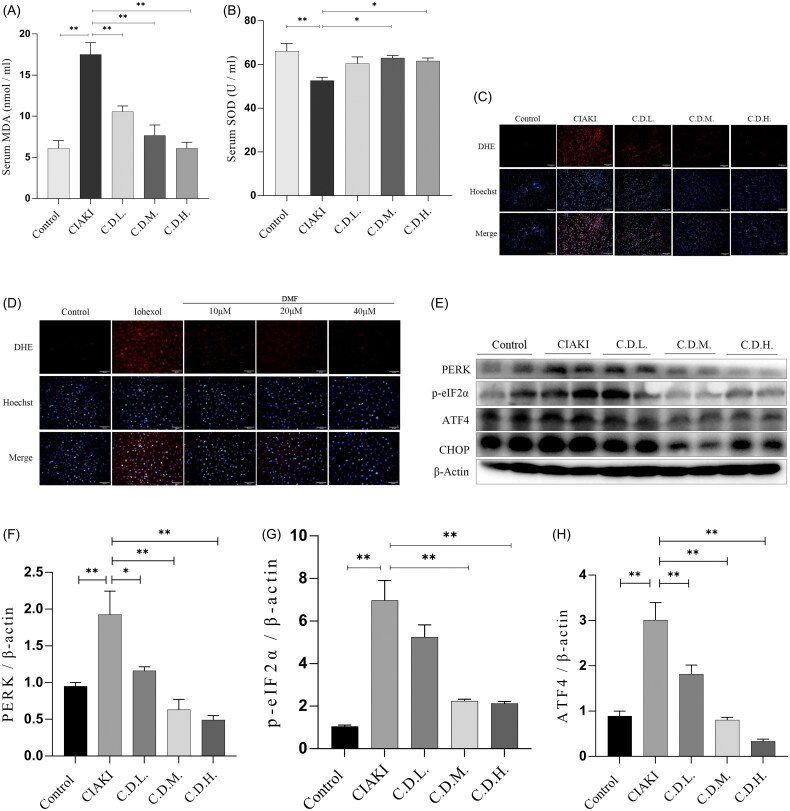

**Figure:**
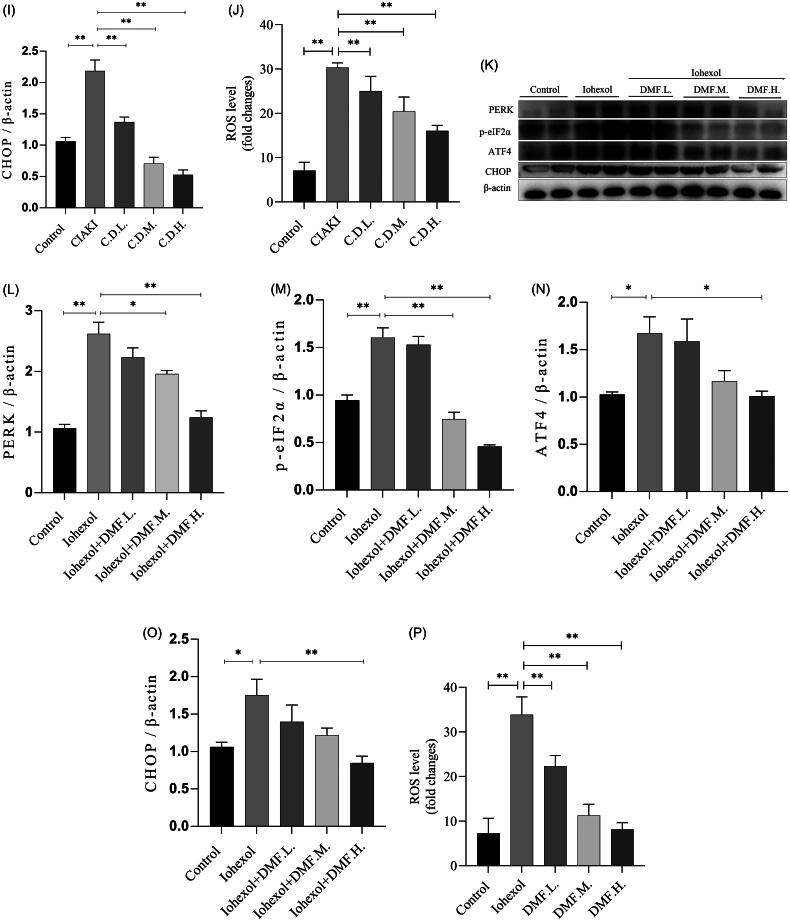

### 4.6. DMF ameliorated pyroptosis through the JAK2–STAT3 pathway in HK-2 cells

Through KEGG pathway analysis, the JAK–STAT pathway was found to be involved in the progression of CIAKI and the treatment of DMF. We further explored whether DMF plays a protective role in CIAKI through the JAK2–STAT3 pathway. *In vivo*, western blot results revealed that the phosphorylation levels of JAK2 (p-JAK2) and STAT3 (p-STAT3) were elevated in the CIAKI group, and this effect was reversed by the administration of DMF (*p* < 0.05, Figure 6(A–C)). *In vitro*, p-JAK2 and p-STAT3 were also increased in iohexol-induced HK-2 cells but decreased after DMF treatment (*p* < 0.05, Figure 6(D–F)). There were no significant differences in JAK2 or STAT3 expression between the control group and CIAKI group *in vivo* or *in vitro*. In addition, STAT3 knockdown ameliorated the increase in p-STAT3 in HK-2 cells induced by iohexol (*p* < 0.05, Figure 6(G–H)). The ELISA and western blot results revealed that STAT3 knockdown reduced the concentrations of IL-1β and IL-18 induced by iohexol in the culture supernatant and the expression of pyroptosis-related proteins (*p* < 0.05, Figure 6(L–P)). The above results showed that DMF can ameliorate pyroptosis through the JAK2–STAT3 pathway.

Figure 6.DMF ameliorated pyroptosis through JAK2-STAT3 pathway in HK-2 cells. (A–C) Representative Western blot images and statistical assessment of p-JAK2 and p-STAT3 in mice. (D–F) Representative Western blot images and statistical assessment of p-JAK2 and p-STAT3 in iohexol-induced HK-2 cells with or without DMF. (G,H) Representative Western blot images and statistical assessment of p-STAT3 in iohexol-induced HK-2 cells with or without siRNA-STAT3 and DMF. (I) Summarized IL-1β and (J) IL-18 concentration in culture supernatant of iohexol-induced HK-2 cells with or without siRNA-STAT3 and DMF. (K–P) Representative Western blot images and statistical assessment of NLRP3, caspase 1, GSDMD, cleaved GSDMD and ASC in iohexol-induced HK-2 cells with or without siRNA-STAT3. *n* = 3. *p < 0.05, **p < 0.01.

**Figure:**
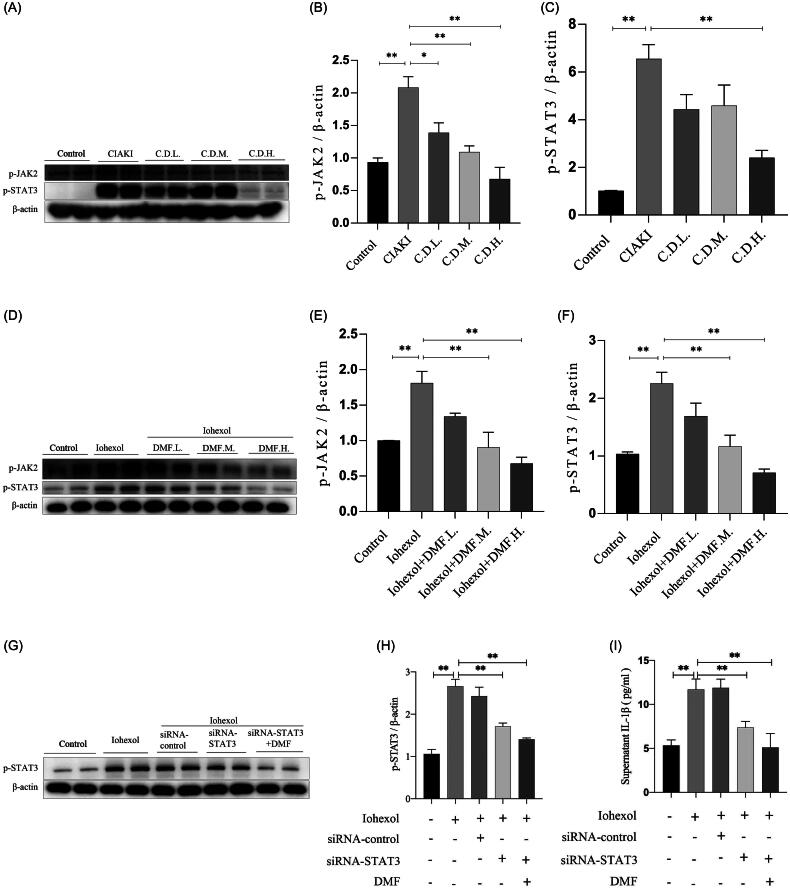

**Figure:**
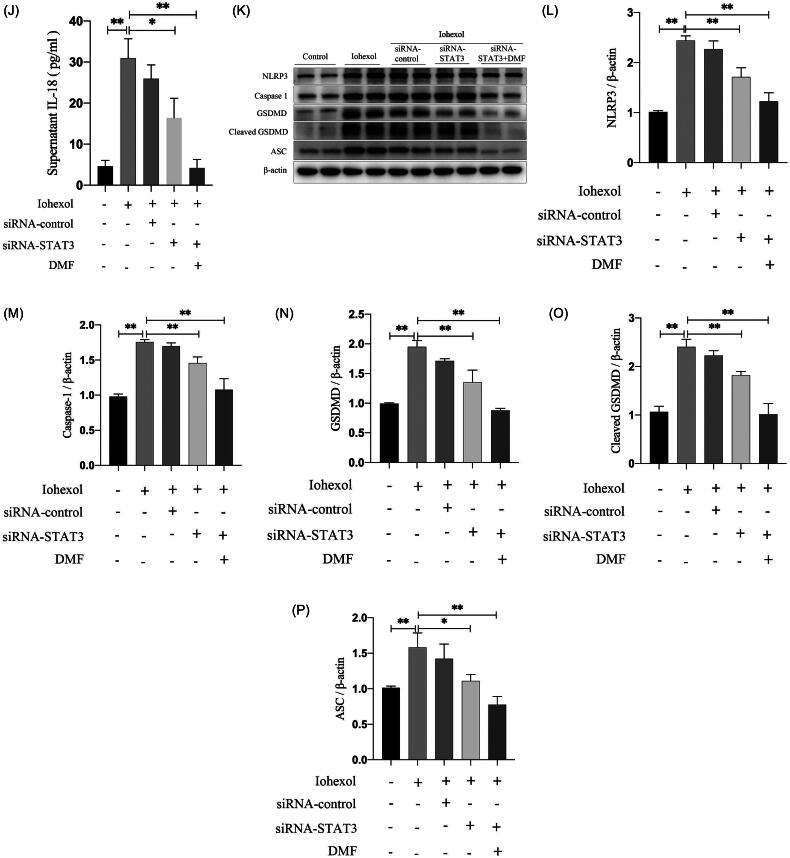

### 4.7. DMF ameliorated pyroptosis by inhibiting ERS and the JAK2–STAT3 pathway

To study the relationship between ERS and the JAK2–STAT3 pathway in CIAKI, HK-2 cells were treated with tunicamycin, an ERS inducer. Western blot analysis revealed that tunicamycin activated the JAK2–STAT3 pathway and that DMF partially reduced it (*p* < 0.05, Figure 7(A–C)). The ELISA results revealed that tunicamycin increased the concentrations of IL-1β and IL-18 in the cell culture supernatant, which was reduced by DMF treatment (*p* < 0.05, Figure 7(D,E)). In addition, tunicamycin promoted pyroptosis-related protein expression, which was decreased by DMF treatment (*p* < 0.05, Figure 7(F–K)). The western blot results revealed that STAT3 knockdown inhibited p-STAT3 expression induced by tunicamycin (*p* < 0.05, Figure 7(L,M)). The ELISA results revealed that STAT3 knockdown reduced the concentrations of IL-1β and IL-18 in the cell culture supernatant induced by tunicamycin and had synergistic effects with DMF (*p* < 0.05, Figure 7(N,O)). In addition, STAT3 knockdown partially reduced the expression of pyroptosis-associated proteins induced by tunicamycin, especially when it was combined with DMF (*p* < 0.05, Figure 7(P–U)). These results indicate that ERS can increase pyroptosis through the JAK2–STAT3 pathway and that DMF ameliorates pyroptosis by regulating ERS and the JAK2–STAT3 pathway in CIAKI.

Figure 7.DMF regulated JAK-STAT pathway through ER stress, improving pyroptosis. (A–C) Representative Western blot images and statistical assessment of p-JAK3 and p-STAT3 in iohexol-induced HK-2 cells with or without DMF and ER stress inducer tunicamycin. (D) Summarized IL-1β and (E) IL-18 concentration in culture supernatant of iohexol-induced HK-2 cells with or without DMF and tunicamycin. (F–K) Representative Western blot images and statistical assessment of NLRP3, caspase 1, GSDMD, cleaved GSDMD and ASC in iohexol-induced HK-2 cells with or without DMF and tunicamycin. (L-M) Representative Western blot images and statistical assessment of p-STAT3 in iohexol-induced HK-2 cells with or without tunicamycin, siRNA-STAT3 and DMF. (N) Summarized IL-1β and (O) IL-18 concentration in culture supernatant of iohexol-induced HK-2 cells with or without tunicamycin, siRNA-STAT3 and DMF. (P–U) Representative Western blot images and statistical assessment of NLRP3, caspase 1, GSDMD, cleaved GSDMD and ASC in iohexol-induced HK-2 cells with or without tunicamycin, siRNA-STAT3 and DMF. *n* = 3. *p < 0.05, **p < 0.01.

**Figure:**
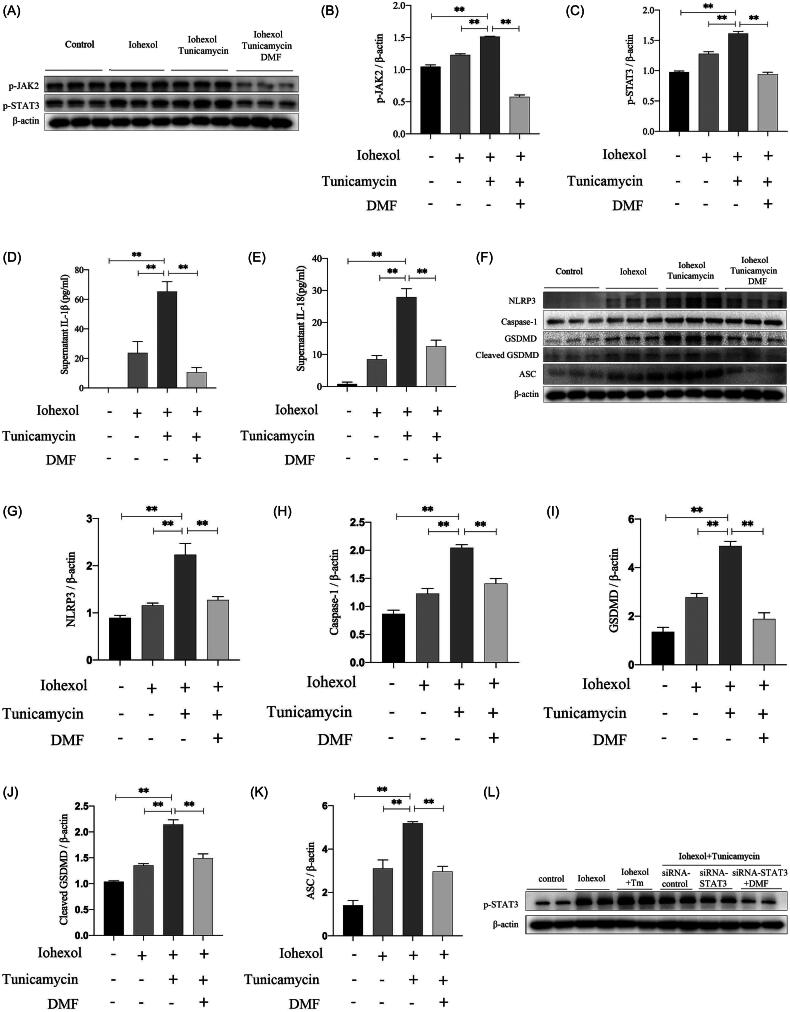

**Figure:**
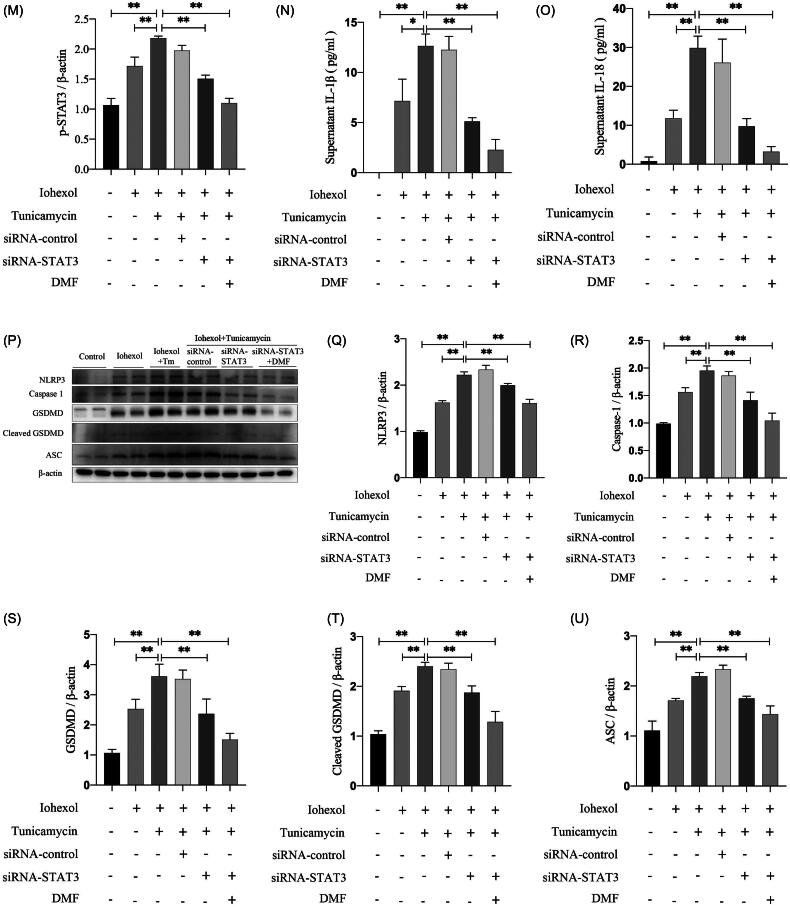

## 5. Discussion

Our study provides the first experimental evidence that DMF significantly attenuates iohexol-induced renal dysfunction in CIAKI patients. To elucidate the molecular mechanisms underlying the therapeutic effects of DMF, we performed RNA-seq analysis on kidney tissues from CIAKI mice. Functional enrichment analysis demonstrated significant enrichment in the acute phase response and JAK–STAT signaling pathways. Pyroptosis is a form of inflammatory programmed cell death [22]. Studies have shown that pyroptosis is involved in the pathogenetic process of CIAKI [9,10]. RCM can increase the expression of Toll-like receptor 4 in renal tissue and renal tubular epithelial cells by increasing the serum S100A8/A9 level and activating the NLRP3 inflammasome, leading to a downstream inflammatory cascade and CIAKI [23]. RCM activated the NLRP3 inflammasome in renal macrophages through renal tubular reabsorption, which led to IL-1β-dependent leukocyte recruitment and induced renal tubular injury, while renal injury in NLRP3^-/-^ mice was mild [24]. DMF has been shown to alleviate pyroptosis in various disease models. For example, in silicosis, caspase-1-mediated cleavage of gasdermin D (GSDMD) and caspase-3/8-mediated cleavage of gasdermin E (GSDME) drive pyroptotic cell death. DMF significantly attenuated this process by inhibiting caspase-1, caspase-3, and caspase-8 activation, thereby preventing GSDMD and GSDME cleavage [25]. However, the effects of DMF on pyroptosis in the context of CIAKI remain unexplored. Our data confirmed that iohexol triggered NLRP3 inflammasome activation *via* JAK2–STAT3 signaling, which is consistent with previous reports of contrast media-induced pyroptosis. DMF treatment effectively suppressed caspase-1-mediated cleavage of gasdermin proteins, mirroring its antipyroptotic effects observed in CIAKI.

The JAK–STAT signaling pathway is an evolutionarily conserved signaling pathway that plays a role in many key physiological processes, including hematopoiesis, differentiation, metabolism and immunomodulation [15]. In the STAT family, STAT3 plays a core role in signal transmission from the plasma membrane to the nucleus, making it a promising target for drug development [26–28]. Multiple studies have demonstrated that JAK2 and STAT3 are essential for inflammation and pyroptosis [29–33] and that DMF can play a therapeutic role in many diseases through STAT3 [34–36]. Studies have shown that the JAK2–STAT3 pathway is overactivated in other types of AKI [32,37–39], and DMF has been shown to play a therapeutic role in several diseases by inhibiting the STAT3 pathway [40–42]. Our study revealed that the JAK2–STAT3 pathway was overactivated in CIAKI and that DMF reduced its phosphorylation level. The JAK–STAT pathway might be related to pyroptosis. The overexpression of STAT1 and STAT3 can promote pyroptosis in astrocytes [43]. In renal diseases, the relationship between the JAK–STAT pathway and pyroptosis is still unknown. We found that STAT3 knockdown improved pyroptosis induced by iohexol in HK-2 cells, indicating that iohexol induced pyroptosis through the JAK2–STAT3 pathway. In addition, studies have shown that there is a relationship between ERS and the JAK–STAT pathway. ERS can activate the JAK1–STAT3 pathway in experimental autoimmune encephalomyelitis, and the activation of STAT3 depends on PERK, which is the core component of ERS. The inhibition of PERK can eliminate the STAT3 activation induced by ERS. In our study, knockdown of STAT3 inhibited the JAK2–STAT3 pathway and pyroptosis induced by tunicamycin, and it had a synergistic effect with DMF, indicating that ERS can induce pyroptosis through the JAK2–STAT3 pathway and that DMF can inhibit pyroptosis through the inhibition of ERS and the JAK2–STAT3 pathway.

In addition, we found that DMF significantly reduced ER stress, which was consistent with its ability to regulate ER stress in other diseases [21,44]. In a multiple sclerosis mouse model, ER stress-induced activation of the JAK1–STAT3 axis led to the expression of interleukin 6 and several chemokines, and the activation of STAT3 signaling was dependent on PERK [45]. In our study, we found that ER stress activated JAK2–STAT3 signaling in CIAKI and that this pathway was potently inhibited by both STAT3 knockdown and DMF treatment. STAT3 silencing abolished iohexol-induced pyroptosis in HK-2 cells, establishing JAK2–STAT3 as a critical mediator of CIAKI. The synergistic effects of DMF and STAT3 knockdown demonstrated that DMF simultaneously targeted both ERS and JAK2–STAT3 signaling to inhibit pyroptosis. This dual mechanism explains its superior efficacy compared with single-pathway inhibitors.

In summary, this study demonstrated that DMF has therapeutic effects on CIAKI. Specifically, DMF ameliorated pyroptosis by inhibiting ER stress and the JAK2–STAT3 signaling pathway. However, this study has several limitations that should be acknowledged. While siRNA-mediated silencing confirmed the role of JAK2–STAT3, future investigations employing pharmacological JAK–STAT pathway inhibitors will be essential to translate these findings into therapeutic applications. In addition, while this study focused on the role of STAT3 in CIAKI, other STAT isoforms warrant investigation. Future studies should explore cell-specific contributions using conditional STAT3 knockout models and investigate long-term renal outcomes in chronic CIAKI patients.

## Supplementary Material

Supplemental Material

## Data Availability

All data that support the findings of this study will be available from the corresponding author upon reasonable request.
