# Supplementary material for: Dimethyl fumarate ameliorated pyroptosis in contrast-induced acute renal injury by regulating endoplasmic reticulum stress and JAK2-STAT3 pathway
Source: Ren Fail. 2025 May 19;47(1):2504633. doi: 10.1080/0886022X.2025.2504633 (PMC12090309; doi:10.1080/0886022X.2025.2504633)
Supplement: Supplemental Material [file IRNF_A_2504633_SM0104.docx]

**Table 1.** Real-time PCR primer sequences for cDNA

| **Target gene** | **Primer sequence** |
| --- | --- |
| GAPDH (mouse) | Forward5'-AAGAAGGTGGTGAAGCAGGCATC-3' |
|  | Reverse5'-CGGCATCGAAGGTGGAAGAGTG-3' |
| GAPDH (human) | Forward5'-CACCCACTCCTCCACCTTTGAC-3' |
|  | Reverse 5'-GTCCACCACCCTGTTGCTGTAG-3' |
| IL-6(mouse) | Forward5′-TAGTCCTTCCTACCCCAATTTCC-3 |
|  | Reverse5'- TTGGTCCTTAGCCACTCCTTC-3′ |
| IL-6(human) | Forward5'-ACTCACCTCTTCAGAACGAATTG-3' |
|  | Reverse5'-CCATCTTTGGAAGGTTCAGGTTG-3' |
| TNF-α(mouse) | Forward5′-CAGGCGGTGCCTATGTCTC-3 |
|  | Reverse5'-CGATCACCCCGAAGTTCAGTAG-3′ |
| TNF-α(human) | Forward5′-CCTCTCTCTAATCAGCCCTCTG-3 |
|  | Reverse5'-GAGGACCTGGGAGTAGATGAG-3′ |
